# Employee-Driven Innovation in Health Organizations: Insights From a Scoping Review

**DOI:** 10.34172/ijhpm.2023.6734

**Published:** 2023-05-15

**Authors:** Stephanie B.M. Cadeddu, Labante Outcha Dare, Jean-Louis Denis

**Affiliations:** ^1^Health Hub: Politics, Organizations, and Law (H-POD), University of Montreal, Montreal, QC, Canada; ^2^University of Montreal Hospital Research Centre (CRCHUM), Montreal, QC, Canada; ^3^School of Public Health, University of Montreal, Montreal, QC, Canada; ^4^Department of Health Management, Evaluation and Policy, School of Public Health, University of Montreal, Montreal, QC, Canada

**Keywords:** Scoping Study, Frontline Employees, Innovative Organizations, Bottom-Up Innovation, Health System

## Abstract

**Background:** Employee-driven innovation (EDI) occurs when frontline actors in health organizations use their firsthand experience to spur new ideas to transform care. Despite its increasing prevalence in health organizations, the organizational conditions under which EDI is operationalized have received little scholarly attention.

**Methods:** This scoping review identifies gaps and assembles existing knowledge on four questions: What is EDI in health organizations and which frontline actors are involved? What are the characteristics of the EDI process? What contextual factors enable or impede EDI? And what benefits does EDI bring to health organizations? We searched seven databases with keywords related to EDI in health organizations. After screening 1580 studies by title and abstract, we undertook full-text review of 453 articles, retaining 60 for analysis. We performed a descriptive and an inductive thematic analysis guided by the four questions.

**Results:** Findings reveal an heterogeneous literature. Most articles are descriptive (n = 41). Few studies are conceptual and empirical (n = 15) and four are conference papers. EDI was often described as a participatory, learning innovation process involving frontline clinical and non-clinical staff and managers. Majority EDI were top-down, often driven by the organization’s focus on participatory improvement and innovation and research-based initiatives. Five categories of methods is used in top-down EDI, two thirds of which includes a learning, a team and/or a digital component. Hybrid EDI often involves a team-based component. Bottom-up EDI emerged spontaneously from the work of frontline actors. Enablers, barriers, and benefits of EDI are seen at macro, organizational, team and individual levels; some benefits spread to other health organizations and health systems.

**Conclusion:** This scoping review provides a comprehensive understanding of the organizational conditions under which EDI is operationalized. It offers insights for researchers, health organizations, and policy-makers about how and why frontline actors’ involvement is crucial for the transformation of care.

## Background

 Innovation in healthcare refers to “those changes that help healthcare practitioners focus on the patient by helping healthcare professionals work smarter, faster, better and more cost effectively.”^1^ The concept of employee-driven innovation (EDI) follows the same tenets, yet leverages the role of “employees close to where services are delivered,”^2^ creates a context and deploys mechanisms in health organizations that can be described as “frontline staff-led”^3^ and “open and collaborative.”^4^ While entrepreneurial doctors have historically driven clinical and technological innovations,^5^ EDI concentrates on the role of a broader set of health professionals, health workers and managers of health organizations that deliver care in shaping health innovations. These innovators are often referred to as “front-line innovators,”^6^ “healthcare insiders”^7^ or “street-level bureaucrats.”^8^ They play a crucial role in the healthcare sector^9^ and their involvement in innovation processes of health organizations can positively impact the quality of healthcare.^10,11^

 Innovations in public and private healthcare sectors are traditionally driven and developed by “upper organizational levels,”^12^ are characterized as top-down and demanding,^2,13^ and are often privileged over other types of innovation.^14^ Top-down processes reflect “a compliancy-driven approach” to innovation,^15^ that may be associated with “the hierarchical nature” of healthcare.^16^ Alternate approaches to innovation are recognized as having an important role to play in leveraging all sources of knowledge within an organization.^17^ For this reason, EDI is encouraged by national and local initiatives to involve frontline actors in healthcare improvement. Examples are the Robert Foundation programme ‘Transforming Care at the Bedside,’^18^ Kaiser Permanente’s Garfield Innovation Center,^19^ the Boston Children’s Hospital,^20^ and the Centre Hospitalier de l’Université de Montreal’s (CHUM’s) organizational and cultural transformation to stimulate bottom-up innovation.

 These endeavors are justified by the many advantages associated with the development of EDI in health organizations. Notably, EDI can address resource constraints in health organizations by allowing frontline workers to engage in processes of “appropriation and repurposing” the resources at hand.^21^ The COVID-19 pandemic has prompted frontline workers to improvise and innovate, for instance, on pedagogy and post-mortem protocols, which enabled greater health system responsiveness.^8,22^ EDI also takes shape in small scale interventions, such as competitions, for rapid discovery and testing of new ideas by frontline employees.^4,23^ As well, support for EDI can encourage frontline actors to become leaders in transforming care,^24,25^ addressing structural inequalities in health organizations.^7^ Subsequently, EDI is increasingly relevant in the context of health systems striving to optimize patient care, professional well-being and health system performance while reducing costs (ie, objectives that refer to the Quadruple Aim framework^26^). More recently, the “Quintuple Aim” promotes the idea of health equity.^27^ This evolution reveals the constant challenge facing health system authorities and micro-system actors to review, adapt and innovate, and emphasizes the importance of EDI in this endeavour.

 There are a number of challenges to creating this dynamic of innovation within organizations to bring about the benefits of EDI.^28^ For example, if rapid discovery competitions lack organizational support, EDI stemming from them tends to result in “short-lived project ideation but no sustainable solutions.”^29^ Local innovations that provide quick fixes without impacting organizational processes^30^ can remain poorly documented and leave the precise value of EDI unclear. EDI stemming from spontaneous and uncoordinated employees’ ideas can be unpredictable, which can undermine the order of the system in place.^31^ Implementing and sustaining innovation in healthcare also remains difficult^32,33^ and the health sector is often considered as a change-resistant environment.^34^ Consequently, when complex organizations like health organizations commit to a new dynamic of innovation, a main challenge is to bring about organizational and institutional change,^35^ and create “…new structures within a given organization[…].”^6^

 Questions remain regarding how to organize and enable EDI within organizations.^31^ There are, as yet, few studies to guide EDI practices and the creation of organizational conditions in which EDI can be operationalized. This scoping review thus seeks to assemble available evidence to inform these efforts in the context of healthcare. An exploratory scan of bottom-up innovation and particularly EDI literature revealed diverse sectors where these concepts have been studied, including the military,^36^ public, and private service organizations^37^ and the health sector.^5^ Studies looking at the structural, organizational, and individual determinants of bottom-up approach to innovation including EDI are rare (eg, ^37,38^). Terms to describe EDI are also diverse, such as “service encounter-based innovation,”^37^ “frontline innovation”^39^ or “employee involvement in … innovation.”^11^

 This lack of consensus on terms, definitions and models in the healthcare context motivated this scoping review. It follows a systematic approach to provide definitional and conceptual clarity to EDI, and broadly map the conditions within which EDI is deployed in health organizations. The exercise also hopes to clarify why it is important for health organizations to support EDI. Scoping reviews are suitable for looking at emerging innovation fields.^40^ This one takes an innovation management perspective, and seeks to answer four questions: (1) What is the definition of EDI and who is involved? (Definition level); (2) What are the characteristics (sources, drivers and methods) of the EDI process (from idea generation to development)? (Process level); (3) What are the contextual enablers of and barriers to EDI? (Contextual factor level); and (4) How does EDI contribute to health organizations and under what circumstances is it beneficial for health systems? (Intra and extra-organizational benefits level). Finally, the review highlights key gaps in the field.^41^

## Methods

 This scoping review focuses on the research questions mentioned above and follows the four steps proposed by the Johanna Briggs Institute^42^: (1) Search strategy; (2) Screening and selection of sources of evidence; (3) Data extraction; and (4) Data analysis.

###  Search Strategy

 The research team began by searching and reviewing some 15 articles to grasp the vocabulary commonly used to refer to bottom-up innovation and EDI. Several trial searches were then undertaken to identify, refine and reduce keywords used in various databases. For example, *bottom-up innovation* was not a popular term, so keywords stemming from the literature on frontline innovation, employee-driven innovation and employee participation were added. The team had the assistance of a librarian specialized in public health, who advised on word iterations and conceptual planning, validated the final set of keywords and verified the resulting list of articles.

 Several combinations of keywords were developed, stemming from three key concepts: bottom-up, innovation and health organization (for more details, see Supplementary file 1, Table S1).^11,16,43^ After testing the keywords in several databases, the final search was undertaken in seven multidisciplinary databases: Proquest Central, EMBASE, Scopus, Business source complete and Academic Search Complete (EbscoHost), Medline and Web of Science (see an example of research equations in Supplementary file 1, Table S2). As no timeframe was indicated when searching for the articles in these databases, studies published from the 1980s to 2019 were included. Article search and selection was accomplished in December 2019.

###  Inclusion and Exclusion Criteria

 Inclusion and exclusion criteria for this scoping review are shown in Table 1. Records included all types of peer-reviewed literature and grey literature. Although various sorts of knowledge could enrich the answer to the research questions, the use of peer-reviewed publications acted as a proxy for science integrity, and ensured that evidence reported by studies were suitable for publication. Languages were limited to English and French, but there were no restriction regarding studies’ country of focus. Other key inclusion and exclusion criteria were based on the three concepts mentioned above: bottom-up, innovation and health organization; they are described below.

**Table 1:** Inclusion and Exclusion Criteria for Selecting Literature

**Criteria**	**Included**	**Excluded**
Type of literature	Peer-reviewed studies, grey literature (such as case studies from Harvard Business Review and government reports), conference papers and proceedings, books and online journals	Conference posters, abstract-only papers, dissertations
Countries and languages	Studies from all low-, middle- and high-income countries written in English or French	Studies written in languages other than English and French
Bottom-up	Studies considering frontline actors as a source of innovation and highlighting their appropriation of innovation projects at least from idea generation to idea development	Studies involving frontline actors temporarily or later in the innovation process such as collecting ideas from frontline actors without involving them in the innovation process
Innovation	Studies considering the innovation process and its methods of innovation, at least from idea generation to idea development; studies considering the innovation outcome defined as a new or an improved change in the organizational context (incremental and radical innovation)	Studies looking at methods, from change management, quality improvement, leadership agency and other literatures, that were not used to generate and develop innovation
Health organizations	Organizations that deliver care, such as hospitals, clinics, mental healthcare facilities, pediatric centres and more, involving physicians, clinicians, nurses, patients and other stakeholders	Studies looking at private organizations such as pharmaceutical companies were excluded

 The concept of ‘bottom-up’ considers two main characteristics: (1) the source of the innovation – frontline actors whose everyday responsibilities are outside innovation, sometimes in collaboration with other stakeholders; and (2) the involvement of frontline actors in the appropriation of projects at the start of the innovation process – at least in idea generation and development activities. This is different from being involved temporarily by participating in one or a few stages of a process.

 The concept of ‘innovation’ can be considered as both a process and an outcome. As a process, innovation refer to the ideation, design or development (production) of a new idea, eventually resulting in the “adoption, assimilation, and exploitation”^44^ of the idea. The resulting innovation can consist of an organizational, technological, social, product/service, practice, system or process innovation. Particular to health innovation, innovation outcomes can be “new or improved health policies, systems, products and technologies, and services and delivery methods that improve people’s health and wellbeing.”^45^ An innovation can thus be considered as an improvement to healthcare professionals’ practices and the organizational environment in which they evolve. Disruptive and non-disruptive innovations as well as incremental and radical innovation are considered. Papers addressing concepts that relate to innovation, such as change management, quality improvement and leadership agency, were excluded when health organizations did not use these concepts to generate and develop innovation. Studies were excluded, for instance, when quality improvement methods served as a tool to collecting ideas from employees without involving them further in the process nor without mentioning notable changes or innovation.

 Finally, ‘health organization’ refers to healthcare providers, that are organizations that deliver care, such as hospitals, clinics, mental healthcare facilities, pediatric centres and more, involving physicians, clinicians, nurses, patients and other stakeholders. Studies looking at private organizations such as pharmaceutical companies were excluded. The quality of the extracted studies was not explicitly assessed according to the criteria of the scoping review approach.

###  Screening and Selecting Sources of Evidence 

 The Covidence software was particularly helpful in screening and selecting articles based on the abovementioned criteria. Duplicates were automatically eliminated. Two of the three authors undertook screening and selection, following a rigorous two-step process informed by PRISMA-ScR (Preferred Reporting Items for Systematic reviews and Meta-Analyses extension for Scoping Reviews).^46^

 The first step involved screening articles based on their title and abstract. Two trial runs were undertaken. In the first, 50 articles retrieved from Business Source Complete were screened to ensure that the two reviewers came to a consensus with the third researcher on which articles to select, thereby refining their understanding of the literature as well as the inclusion and exclusion criteria. This is a key step to reinforcing the rigor of a scoping review.^47^ Once consensus was achieved between the three reviewers on that sample, another sample of 100 articles retrieved from the Scopus database was screened to see whether inter-reviewer differences diminished, which was the case. This allowed the two reviewers to proceed with screening the titles and abstracts of the remaining articles. Full-text paper was retrieved if the abstract did not contain enough details to decide whether to include it. A total of 453 articles were retained.

 The second step involved screening the full text articles retained in the previous step. The three authors read 10 randomly selected articles to achieve consensus on what articles should be included in the scoping review. Once agreement was reached, two reviewers then screened the 453 full-text articles, based on the previously mentioned inclusion and exclusion criteria, along with a further exclusion criterion: articles that mentioned EDI but did not provide information related to any of the scoping review’s four research questions. A total of 58 articles were retained at this stage. Two articles identified in references of other papers were added, for a total of 60 articles.

###  Data Extraction 

 A data chart was developed to ensure that researchers extracted similar information from the 60 studies around the four research questions. An Excel file was created to record information pertaining to three broad domains: (1) the article citation (authors, title, year), methodology, objectives, country and health organization targeted (when applicable) and nature of the article categorized as either of the following three categories: descriptive (*a*. without evaluation of the intervention; *b*. with poor evaluation [eg, measures only satisfaction]; *c*. with strong evaluation [eg, using quantitative or qualitative methods]), analytical (*a*. theoretical or *b*. theoretical and empirical) and conference paper; (2) the EDI topic majorly addressed in the article categorized as one or more of the four research questions; and (3) quotes pertaining to the four research questions. This data chart was modified in research team meetings several times throughout the extraction process to achieve consensus on the extracted data.

###  Thematic Data Analysis

 The 60 full text papers were analyzed based on the data chart described above, using inductive thematic analysis. The four research questions were used to create four level of analysis: (1) definition of EDI in health organizations and the type of frontline actors involved, (2) the characteristics of the EDI process, (3) contextual factors such as the system, organizational, or individual enablers and barriers that underpin the emergence and development of EDI, and (4) EDI benefits. This study did not inventory EDI outcomes, but rather sought to understand how the body of research defines EDI and maps EDI conditions of success in health organizations. Based on an initial reading of the extracted studies, three common elements of the EDI process were identified: the source of the EDI process (ie, top-down, hybrid or bottom-up),^48^ the driver behind the initiation of the EDI and the key methods to operate the EDI process (from idea generation to development). We did not focus on each method used for each stage of the process, such as when brainstorming for idea generation was cited. Also, a study could report more than one method.

## Results

###  Scoping Review 

**Figure 1:**
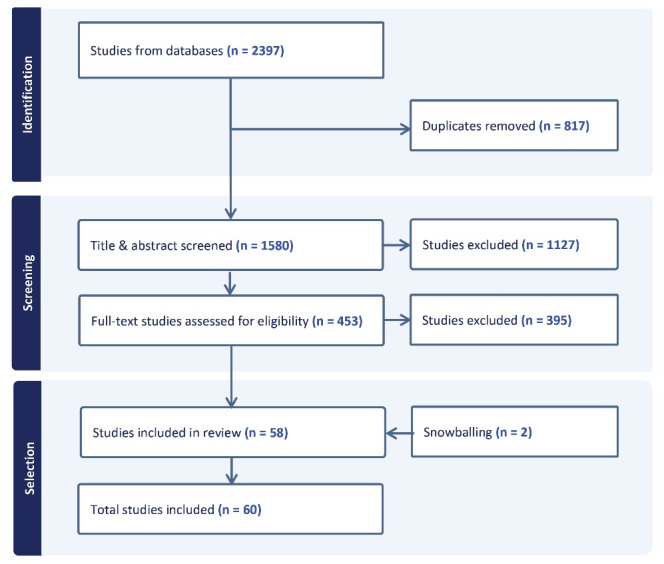
PRISMA-ScR Screening and Selection Process. Abbreviation: PRISMA-ScR, Preferred Reporting Items for Systematic reviews and Meta-Analyses extension for Scoping Reviews.

Figure 1 exhibits the PRISMA-ScR screening and selection process. We extracted 2397 articles from which 817 duplicates were removed. The title and abstract of 1580 articles (identified using the search strategy described above) were screened, and 1127 were excluded as not relevant, leaving 453 articles eligible for full-text screening. Of these, 395 were excluded for the following reasons: 169 were not relevant, 149 did not primarily address EDI, 45 did not fit into one of the included categories of articles (eg, conference abstracts, non peer-reviewed articles), the full text was unavailable for 16, 14 were duplicates, and two were in a language other than English or French. Snowballing led to the addition of two articles, leading to a total of 60 articles.

 Of the 60 articles, 41 were descriptive, 15 were analytical, and four were conference papers (see Supplementary file 2 – Table S3, Table S4, and Table S5^3,11,12,15,16,20,24,25,35,39,43,49-97^ for an overview of the extracted literature).Descriptive articles referred to non-theoretical empirical case studies published in peer-reviewed journals. The descriptive studies included no evaluation of the EDI initiative (n = 21), while others included evaluation that ranged from poor (n = 8) to strong (n = 12). Descriptive studies focused mainly on the EDI process (n = 31), with some articles also exploring enablers (n = 14) and benefits (n = 7). Analytical articles published in peer-reviewed journals were either strictly conceptual (n = 2) or both conceptual and empirical (n = 13), relying on primary research using qualitative, quantitative or mixed methodologies. Analytical studies focused mainly on enablers of EDI (n = 11); only five looked into the EDI process and three explored EDI benefits. Finally, conference papers only targeted the EDI process (n = 3) and enablers (n = 2).

 Of these 60 articles, the journal in which the most studies were published was *Nursing Administration Quarterly* (n = 4), followed by *Harvard Business Review* (n = 3), *Journal of Advanced Nursing* (n = 3), *Nursing Management* (n = 2), the *Joint Commission Journal on Quality and Patient Safety* (n = 2), *European Journal of Innovation Management* (n = 2), *Journal of Nursing Management* (n = 2) and *Journal of Nursing Administration* (n = 2). However, these journals were not representative of all fields in which studies were published, as we found 36 different journals that were mostly highly specialized or professionals. Of these, nursing and healthcare topics were the most represented, followed by innovation management and information system fields.

 The predominant jurisdictions where studies were conducted were the United States (n = 26), distantly followed by Canada (n = 4), the UK (n = 4), Norway (n = 4), Australia (n = 3), Netherlands (n = 2), Italy (n = 2), Israel (n = 2) and Finland (n = 2). Only three studies related to middle-income countries, including Brazil,^82^ Pakistan,^60^ and Thailand^49^ and no studies focused on low-income countries. Three studies were theoretical or did not relate to a specific jurisdiction (ie,^53,58,87^). Overall, the health organizations studied were most often hospitals and medical centers (n = 46), including six academic health centres, distantly followed by care homes (n = 4) and clinics (n = 3). More than half of the studies were published in 2015 or after.

###  Thematic Analysis

 Few of the studies were analytical, however information was extracted to appreciate the scope of EDI definitions, processes, enablers/barriers and benefits discussed by the broader literature. It is important to remember that the prominence of each of these can fluctuate from one context to another and thus results are not generalizable to all health organizations, units, or medical sectors.

###  Definitions of Employee-Driven Innovation

 To answer the first research question, two aspects were isolated: definitions linked to EDI and frontline actors involved in EDI. While 42 articles offered specific boundaries to the concept, they did not offer a formal definition of EDI. As no two definitions of EDI were the same, we assembled the variety of definitions into three dimensions, based on an inductive analysis: EDI as a participatory innovation process, EDI as a learning process, and EDI as an innovation outcome. Examples in each of these dimensions are presented in Table 2. Although the extracted definitions sometimes fell into several categories, classifying dimensions highlight differences between their key focus.

**Table 2:** Samples of Employee-Driven Innovation Definitions

**Dimensions**	**Sample of Definitions **	**Study**
Participatory innovation process (ideation, development and implementation)	*“All of the staff in the hospital were involved both in defining the problem and in the efforts to seek and develop appropriate organizational solutions”* (p. 233).	^90^
	*"Engagement of bedside nurses to transform work processes, quality of care and staff satisfaction on medical-surgical units"* (p. 669).	^52^
	*“Adopting a bottom-up approach to identify priority problems and the changes that may be needed”* (p. 7).	^58^
	*“Approach to involve all stakeholders in the conduct of projects”* (our translation) (p. 28).	^63^
Learning process (learning, mentoring, harnessing employees’ innovative capacities)	*“Bringing frontline staff to the table provided an opportunity to engage and mentor nurses while solving problems … [;] teaching through doing was the best approach to engage and mentor frontline staff”* (p. 70).	^66^
	*“Employees with IWB [Innovative Work Behaviour] can quickly and appropriately respond to customers, propose new ideas and create new products”* (p. 155).	^49^
	*“Engaging and educating staff within the clinical microsystem and building the capacity for rapid-cycle improvement at the unit level” *(p. 445-446).	^72^
	*“Engaging and empowering front-line primary care teams with multi-level, interdisciplinary stakeholders in structured EBQI [Evidence-based Quality Improvement], and facilitated provider and staff initiated innovation projects”* (p. 3).	^83^
Innovation outcome	*“New ideas and design products and procedures based on the employees’ own observations” *(p. n.a.).	^94^
	*“Grassroots improvement ideas from frontline clinicians”* (p. 141).	^68^
	*“Ward creating solutions at the point of care” *(p. 466).	^67^

*Note*: n.a. stands for not available.

 To involve employees in the innovation process, the first dimension designates a participatory component in the identification of problems, the generation of new solutions, followed by their development and implementation in frontline actors’ work context (eg,^25,62,93^). Studies explicitly reported the importance of collaborating with either a minority, or some or all employees in innovation efforts. In the dimension of EDI as a learning process, definitions placed a strong emphasis on empowering frontline workers to innovate through learning, discovering, and testing new ideas (eg,^20,78^). EDI can be useful to frontline actors as they can obtain mentoring as well as leverage their innovative capacities or “innovative work behavior” (eg,^49,60^). Finally, the dimension of EDI as an innovation outcome focused on the product, service, process, practice or organizational innovation resulting from frontline actors’ ideation and innovation development. These were variously called “bottom-up solutions by frontline providers,”^16^ “solutions at the point of care”^67^ or “frontline staff-led improvement efforts.”^3^ Examples of EDI outcomes include new “policy on siblings visiting the labour ward after a delivery,”^51^ the redesign of a nursing-staff schedule,^65^ the development of pain boards,^52^ or process redesign around chemotherapy delivery.^89^

 In addition to these three definitional dimensions, the analysis extracted details on the type of actors involved in EDI. In all studies, it was clear that EDI stemmed from a single employee or the joint efforts of two or more employees or collaborators who generated, developed and implemented new ideas. Three key findings emerged regarding frontline actors involved in EDI in health organizations.

 First, the majority of studies reported both clinical and non-clinical staff members; clinical staff are direct care staff, whereas non-clinical staff refer to actors involved in a patient’s trajectory of care who are not involved in clinical work. The most frequently reported frontline staff were nurses, physicians, administrators, caregivers and unit clerks. Other staff also involve in EDI include “hospital employees from the IT department and the Kitchen section to different departments within Medicine, Surgery and Health-care”^94^ as well as “cleaning ladies.”^70^

 Secondly, frontline actors are not only those providing patient care; managers of frontline staff, along with other middle managers also figure in groups involved in EDI (eg, “[n]urse executives and bedside nurses”^52^). According to Høyrup,^98^ the focus of EDI is on ordinary employees at all organisational levels who are not tasked with innovation activities but who yet have valuable contributions to make. While frontline actors drove innovation projects, management also initiated projects that enabled frontline employees to innovate.^57,67,79^ Shiparski and Authier^87^ define frontline managers in healthcare as “the keepers of culture, the gateway to evoking a grassroots intelligence network, and they hold a pivotal role in advancing innovation at the point of care.”

 Finally, the groups involved in EDI also often include other stakeholders such as patients and family caregivers, university staff, senior hospital management, university students and external stakeholders such as members of trade unions (eg,^79,80,90^). The choice of which staff and stakeholders to involve in EDI depends on the area of improvement and innovation an organization hopes to pursue.

###  Characteristics of the Employee-Driven Innovation Process

 Regarding the second research question, in the 43 studies that described the EDI process, three characteristics were identified: sources and drivers of the initiative, and key methods used. Studies that were informative but not specifically focused on the EDI process were included, however studies that only briefly described the process were not.

####  Sources and Drivers of Employee-Driven Innovation

 The source ofEDI was most often *top-down*(n = 32 studies), following a management decision to undertake an initiative. Top-down EDI’s drivers included the organization’s desire to involve employees and use their knowledge to foster improvement and innovation (n = 16) (eg,^50,88^). For example, Eriksen and Tollestrup^94^ reported a Danish hospital’s implementation of an “Employee-Driven Innovation in the Health Care sector” initiative. Another driver was research-based initiatives (n = 10) (eg,^35,51^). These were most often launched by upper management or outside researchers; the latter were considered top-down as we assumed they required the approval of senior management (and ethics committee). Other top-down EDI were initiated to address challenges and ultimately improve care (n = 6). For example, Rotenstein^20^ described how “increasing financial pressures, fluctuating payment models, and an increasing prevalence of clinician burnout” (p. 1) led to the development of “an internal investment program […] to improve care and reduce cost” (p. 3).

*Hybrid EDI*(n = 7) were characterized by bottom-up initiatives launched by frontline actors (managers, staff, researchers), that were formalized by upper management early in the innovation process (eg,^15,86^). All studies except one reported that employees initiated the process to address a specific problem and improve care (n = 6). At the Montreal Children’s Hospital, for instance, three physicians observed that the lack of communication between patients and carers could be improved with a patient safety campaign. To move ahead, they sought the “green light by the executive of the hospital.”^68^ One study was driven by a researcher employed at the health organization.^82^

 When EDI was *bottom-up* (n = 4), initiatives emerged from the work of frontline actors in an unstructured or spontaneous way to improve a situation. In this context, the organization did not plan or allocate resources for EDI. For example, two information technology employees worked “under the radar”^96^ to enact an infrastructural innovation for patient communication, which spread across the organization over the next 10 years. Similarly, Knoff,^25^ a telephone triage nurse in a pediatric hospital, understood that parents managed their children’s care differently at the hospital than at home. She thus developed a tool to help parents time their child’s pain medication, showed it to colleagues, and only later approaching the hospital pain committee. Her innovation was implemented 6 years later in the hospital.

####  Methods of Employee-Driven Innovation

 Several methods to operationalize the EDI process were used and more than half the studies mentioned learning, team and digital components (see Supplementary file 3, Table S6, Table S7, and Table S8).^3,15,20,24,25,35,43,50,51,55,59,61,63-65,68-73,75,77,78-83,85,86,88-97^

 Five categories of method were reported when the source of EDI was *top-down*. Studies mentionned *participatory approaches* (n = 10), related to social sciences methods such as focus group,^92^ or a “deliberative approach” offering “a space of debate.”^35^ Most EDI driven by research employed a participatory approach. Other studies mentioned *design tools*(n = 8) such as user-centered collaborative design^63^ or design-thinking (eg,^65,71^). Studies also described use of *competition-based approaches* (n = 7), such as innovation tournaments (eg,^43^) and grant-funded initiatives (eg,^20^) to encourage staff to develop and submit ideas. This method entails financial and intellectual support to materialize employees’ innovation proposals once ideas are selected. Mentioned less often were *quality improvement methods* (n = 5) (eg,^3,72,93^) such as rapid-cycle improvement tools (eg, Plan Do Act Study cycle) and Knowledge Translation Toolkits, as well as techniques such as social entrepreneurship (n = 1)^24^ and change management (n = 1).^69^

 Three components structured methods used in top-down initiatives. There was a *learning-based component* (n = 10), which integrated an ‘innovating while learning’ element in the EDI process (eg,^77,66,94^). All methods, except the competition-based method included a learning component. For example, a children’s hospital in the United States launched two pilot “Innovation Units” to train both managers and frontline staff to lead and develop “local improvement portfolios” using quality improvement tools.^72^ Also, all five categories of methods included a *team-based component*(n = 7) to tap into the collective intelligence of employees. While many methods (eg, participatory approach, design tools) naturally involved forming groups, these studies reported deliberate establishment of a group dedicated to innovation efforts. For example, a “frontline innovation group”^66^ which “provided a forum to enhance empowerment … while seeking solutions to operational failures” (p. 75). Finally, a *digital component*was mentioned (n = 6) to promote the collection, brainstorming and visibility of employees’ ideas (eg,^51,75^). For instance, a touchscreen computer was developed as “a platform for workers’ findings, initiatives, ideas and solution proposals to develop services”^97^ or provide a platform for discussion (eg,^51^).

 For *hybrid EDI processes*, only *participatory* (n = 2) (eg,^61,82^) and *quality improvement methods*(n = 1)** (**eg,^64^) were reported. Studies unrelated to methods mentioned *learning-based*(n = 1) (eg,^68^) and *team-based components* (n = 5) along with an inductive process, such as the creation of a “Nursing Practice Committee as a vehicle for staff involvement in planning and implementing change.”^73^ Looking at *bottom-up EDI*, one descriptive study^78^ recommended a team-based component along with the use of quality improvement tools to increase clinical staff analytical and change capacities. Two studies (ie,^55,96^) described more experimental innovation processes; Knoff^25^ associated the experience with social innovation theory.

 Only a few studies reported the duration of EDI initiatives (from idea generation to implementation); top-down EDI processes were often completed between six months or less and two years; hybrid processes took between 1 to 3 years, and bottom-up EDI after 6 years.

###  Contextual Enablers and Barriers of EDI

 Concerning the third research question, enablers of and barriers to EDI in health organizations were found at three levels: macro, organizational and individual. No particular recipe for enabling EDI within health organizations emerged. Also, enabling and hindering factors were generally intertwined, affecting each other before, during and after the innovation process. As an example, promoting collaboration between staff and management (organizational level) enabled individuals to develop trust in their organization (individual level). Across the three levels, 15 enablers were extracted from 58 studies, while 11 barriers were identified in 23 studies.

 Macro-level enablers and barriers refer to the various actors and forces that prompt health systems, health organizations and individuals to innovate. Four *macro-level enablers* were identified. Health system programs (n = 5) sought to drive EDI at local or broader level. Four studies in this category were evaluations of the Transforming Care At the Bedside program (eg,^52,67^), while one focused on Unit-Based Training.^79^ Other macro-level enablers included government initiatives (n = 2) such as South Australia’s “State policy initiative on improving care of older people,”^93^ educational institution’s innovation curriculum (n = 1) and private and government funding (n = 1). Only one *macro-level barrier* was identified: government’s changing commitment to valued-based reimbursement had a direct influence on the financial priorities of a health organization’s incubator, limiting the scope of its innovative initiatives.^20^

 Organizational enablers and barriers reflect health organizations’ support for or (unintentional) obstruction of innovation initiated by frontline actors. Six *organizational enablers* were identified: the availability of organizational resources (n = 24), organizational culture supporting frontline workers’ creativity, entrepreneurial thinking and willingness to improve their workplace (n = 24) and management support (n = 22). For instance, providing frontline actors with resources could require the allocation of human resources (eg,^88,93^), funding (eg,^63,89^) or time for innovation (eg,^72^), or investing in training of frontline actors (eg,^81,87^). Also, systems that facilitate and stimulate EDI were a noteworthy organizational enabler, found in 18 studies. Systems to support EDI involved eliminating barriers to EDI, such as bureaucracy, hierarchy or fear of risk-taking (eg,^25,87^); creating a dedicated department for EDI such as an innovation laboratory or incubator (eg,^20,63^); or senior management partnering with a design company to initiate EDI (eg,^70,97^). Another organizational enabler referred to mutuality between employer and employee values, goals and collaboration (n = 9) (eg,^12,97^). Lahtinen et al^97^ revealed that frontline actors were more willing to be involved when the EDI process was “…based on meaningful and practical frames which matter to healthcare professionals.”

*Organizational barriers* were mainly the absence of enablers, such as a lack of resources (n = 10), mutuality (n = 3), managerial support (n = 2) and EDI support system (n = 1). Mutuality was lacking when there was disharmony between organizational, employees and managerial goals and values, and poor responsiveness to employee frustrations (eg,^11,49^). Other organizational barriers were a lack of multi-disciplinary teams (n = 2) and ill-adapted incentives for EDI iniatives (n = 2) (eg,^43,50^).

 Individual enablers and barriers represent frontline workers’ personalities, capacities, values and attitudes, which influence their approach to innovation. Five categories of *individual enabler* were identified. The most mentioned (50% of the individual enablers) related to a*proactive personality*(n = 15), reflecting an individuals’ capacity to recognize problems and drive EDI efforts (eg,^39,55^). This personality trait is also described as self-leadership (eg,^54,56^), innovation champion (eg,^12,57^), and commitment to EDI projects (eg,^52^). Innovation champions can be informal and self-nominated, implying that they “initiat[e] and implement […] innovation beyond the direct, routine treatment of the patients he or she is responsible for.”^12^ While their position does not specify innovation, they initiate improvements in their environment without waiting for the organizations’ support. Four other individual enablers of EDI were a favorable work context (n = 5) (eg,^60^), such as support from co-workers and management and boundary integration in their work environnement^60^; frontline actors’ feeling of entitlement (eg, gender, empowerment) to being involved in innovative activities (n = 4) (eg,^50^), their capacities for innovation (n = 4) (eg,^15^) and willingness to share knowledge among colleagues (n = 2) (eg,^49^).

 The four *individual-level barriers* identified show that staff can become reluctant (n = 12) to participate in EDI initiatives. They may lack confidence in their own leadership, be afraid of taking risks or of a lack of buy-in from the organization (eg,^20,89^). The attitudes of managers can impact staff participation in new EDI projects (eg,^71,80^), leaving them feeling abandoned early in the innovation process.^71^ Other barriers were a lack of information for staff on EDI projects (n = 3) (eg,^89^), and their feeling of doing extra-work (n = 3) (eg,^68^).

###  Benefits of Employee-Driven Innovation 

 The final research question concerned benefits resulting from EDI initiatives. These were classified according to similarities in the level at which benefits appeared.

 Intra-organizational benefits are positive proximal consequences of an intervention and can occur at three levels: organizational, team and individual. At *organizational level*, we observed six types of benefits. Changes in institutional and organizational practices (n = 19) were the most mentioned, such as creation of new care spaces, organisational structures, environments, objects, ways of doing things (eg, methodology) and their dissemination (eg,^35,63,69,75^). This type of benefit decreased resistance to change (eg,^73^) and to the integration of EDI into hospital practices as a standard (eg,^25^). Second, the effiency, productivity and improvement of practice (n = 11) involved interventions that changed the staff work habits (eg,^50,55^). A third benefit (n = 11) was improved quality and safety of care. Additionally, EDI brought opportunities for future innovation in the organization (n = 9) (eg,^65^). The least mentioned benefits were cost savings (n = 4) (eg,^76^) and the sustainability of an innovation over time (n = 4) (eg,^75^).

 Team level benefits refer to the advantages an intervention offers to a group. *At this level*, we observed that EDI fostered closer collaboration (n = 11), increasing interprofessional collaboration across departments (eg,^70^), and collaboration between project teams and other programs to gain experience that they could apply to future improvements (eg,^75^). It could also mean involving staff from other health disciplines and managers in solving problems (eg,^15^) by sharing better ways of conducting frontline activities.^81^ A second team-level benefit was a team approach to problem solving (n = 2), which stimulated discussion, innovation and changed the way employees saw and solved problems by creating a sense of common purpose (eg,^39,85^).

 Finally, individual level benefits to frontline staff and patients were grouped into three categories: staff improvement including capacity building, leadership, confidence, self-esteem and meaning creation (n = 18); and staff satisfaction (n = 6) and patient satisfaction and trust (n = 6). For example, employees had a greater sense of belonging to the organization, and more specifically the EDI intervention, and of being appreciated for their ideas and participation (eg,^43^). Moreover, as frontline employees were involved in the experimentation and implementation of changes in their units, there was an increase in their vitality, satisfaction (eg,^3^) and empowerment (eg,^39^). Similarly, several studies showed that patient satisfaction and confidence improved after EDI interventions (eg,^58,84^).

 While our research questions concerned health organizations, nine studies mentioned broader benefits such as the transferability of the innovation project (n = 6) and the diffusion of innovation and creation of further opportunities for innovation in the health system (n = 5). For example, nurses redesigned the procedure for shift changes, an innovation that spread to several departments and was implemented across the Kaiser system.^65^ Similarly, a new hospital-patient communication platform was then rolled out to other departments and eventually other hospitals in Norway.^55,96^

## Discussion: The Conditions of EDI in Health Organizations

**Figure 2:**
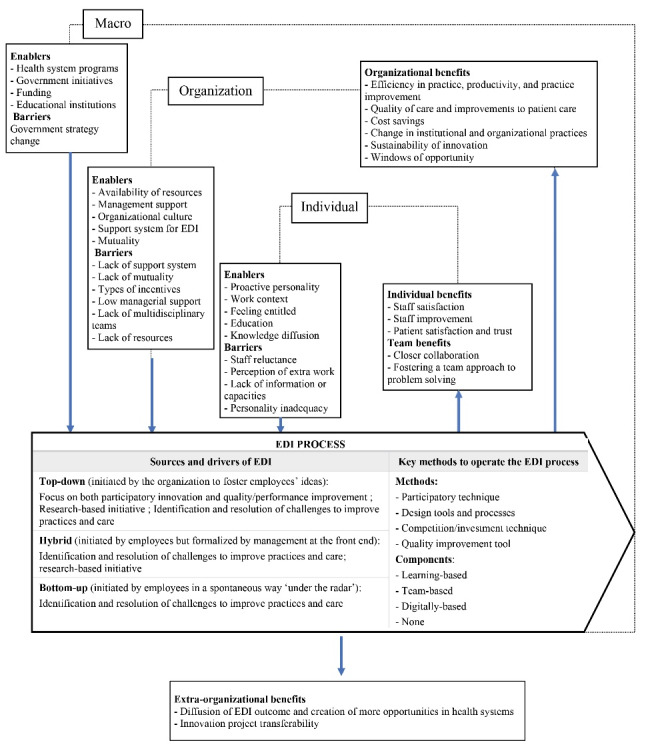
Synthesis of Employee-Driven Innovation Conditions in Health Organizations. Abbreviation: EDI, employee-driven innovation.

Figure 2 proposes a map of the conditions for EDI in health organizations, that is the processes, enablers, barriers, and benefits of EDI found in the literature. This map is a non-normative guide, representing a simplified version of the heterogenous, dynamic and complex reality of innovation management practices.^99^

 This section summarizes findings on the four research questions, and discusses research gaps and opportunities. Starting with the first research question on EDI definition, organisations have different views of EDI and who should be involved. EDI in health organizations emerged as a participatory and learning innovation process leading to innovation outcomes developed by frontline clinical and non-clinical staff and managers. This is similar to EDI in non-healthcare contexts, as EDI “can emerge from “ordinary” employees, from shop-floor workers and professionals to middle managers across the boundaries of existing departments and professions.”^31^ Additionnally, findings show that EDI in health organizations include diverse stakeholders in patient care trajectories.

 To respond to the second research question on the EDI process’ characteristics, three sources of EDI emerged and aligned with the broader literature^48^: a bottom-up process, a mix of top-down and bottom-up processes, and a top-down process. The findings that most EDI were initated top-down suggest that upper management and scholars increasingly recognize the significance of frontline actors in the transformation of care. Hybrid and bottom-up EDI processes were often initiated by frontline staff and managers experiencing problems. In top-down EDI processes, employees responded to leadership expectations and demands, rather than issues they experienced directly in their workplace.

 When organizations adopted top-down approaches to encourage bottom-up innovation, participatory, design-led, competition, and quality improvement methods aimed to increase employee adherence and participation, and organize the development and implementation of ideas. Learning-based components gave employees the autonomy to apply what they learned from the EDI initiative to their everyday job and thus enhanced employee capabilities and willingness to address issues they faced.^98^ Methods that enhance employee innovative capacity can sustain EDI efforts in the long run.^49^ The prevalence of team-based components illustrated the collective nature of EDI. Given the plurality of leadership in innovation teams, and the dynamics of collaboration (eg, designation of formal and informal team leaders), their impact on the effectiveness of team-based EDI methods could be however better understood.^100^ Teglborg-Lefèvre^101^ suggested exploring the multiple strategic intentions and modes of regulation of social relations employed by organizations to support and sustain EDI practices. Finally, few studies on top-down EDI concerned technologies to stimulate or enable EDI emergence and development,^102^ despite the growing presence of digital health and artificial intelligence in health organizations.

 Bottom-up EDI methods involved what ressembles ‘bricolage,’ a creative technique leveraging ongoing interactions with users as well as resources at hand to address particular needs in the workplace. Organizations and frontline actors might benefit from combining bricolage with management-driven (top-down) or management-mediated (hybrid) processes.^103^ Employees who directly experience situations that need to be improved could benefit from managers’ knowledge and competencies in framing and supporting their innovation efforts. Solutions stemming from employees have demonstrated their effectiveness in some urgent contexts when resources become scarce (eg, COVID-19^8^); it would thus be interesting to explore how top-down methods can enable more hybrid and bottom-up EDI to address more immediate issues confronting frontline actors.

 Looking at the third research question, macro level contextual enablers and barriers were poorly explored despite the fact that reforms and government pressure exert considerable influence on the management and organization of innovation broadly and particularly of EDI. An important knowledge gap remains around macro-level factors supporting health organizations’ EDI efforts. This is in line with Chaudoir and colleagues’^104^ systematic review, which found that studies of implementation frameworks rarely reported on structural factors (eg, policies, socio-economic context, local infrastructure). Policy-makers must remain responsive to the needs of health systems, and the findings of this scoping review may help them decide whether and how to support EDI in health organisations (eg, through funding policies or skills development^77^).

 The organizational enablers we find are similar to traditional determinants of innovation.^13,105^ Establishing a support system for EDI appeared as an enabler that particularly attested to an organization’s readiness to change and willingness to eliminate structural and administrative barriers to EDI “beyond what might be accomplished by programs that foster innovations in individuals.”^106^

 The proactive personality of employees with a problem-solving orientation was recognized as a driving force and such people are a valuable resource for hybrid and bottom-up EDI. Management’s role in supporting social relations and the apparent benefits of genuine interpersonal efforts to increase frontline staff confidence in the leadership team (eg,^18,97^) are in tune with Hansen and colleagues’^107^ empirical findings in a study of 20 Norwegian organizations that successfully upheld EDI initiatives. For these authors, “managers should adopt an informal and cooperative style of leadership.”^107^ Exploring different modes of coordination may also address agency-related problems,^101^ which touch upon “authority to make decisions about innovations” in organizations, and the “extent and level of the […] decision rights”^31^ for employees and managers. In this endeavor, Saari et al^108^ raised the important role played by middle and top managers in facilitating a “bridging agency” during the EDI process to respond to agency-specific issues. It seems evident that macro, organizational and individual cannot be considered in silos; studies should explore their interwoven impacts at different stages of the EDI process.

 Looking at the fourth research question, findings on the intra-organizational benefits of EDI in health organizations were sparse but align with the Quadruple Aim in healthcare, a framework at the forefront of health sytems reforms to optimize patient care, professional well-being and health system performance while reducing costs.^26^ Morever, some benefits extended beyond organizational walls. Further analytical research is yet needed. This finding supports Lehoux and colleagues’ international scoping review of health-system challenges, which highlights the sparsity of studies on health innovations supporting health system. This is surprising given the number of existing EDI efforts identified in this scoping review, the health system’s need for health innovation^109^ as well as the role of frontline innovators in health system performance.^33^ Given the discrepancies between government reforms and local organizations’ context and challenges,^110^ exploring how an EDI initiative benefits health systems would help better aligning macro-level support.

 This scoping review reveals knowledge gaps and opportunities for further research. Despite the significant role employees play in driving innovation, EDI in health organizations is still understudied, which is consistent with findings about EDI in more general contexts.^38,111^ Studies included inconsistent or poor information on the cost and time required to undertake an EDI initiative in health organizations as well as on idea selection and evaluation.^111^ Most studies provided snapshots of an EDI initiative, with cross-sectional description and evaluation of EDI activities. Longitudinal research would offer greater insight into how EDI initiatives evolve and are sustained through time. The high proportion of studies published in journals related to nursing attests to greater recognition of the role nurses play in the transformation of care (eg,^112,113^), though studies rooted in innovation management contributed robust analysis of individual enablers of EDI. Interdisciplinary studies have a contribution to make to this stream of literature.^111^

###  Strengths and Limitations

 To our knowledge, this review is the first attempt to map the conditions that underpin EDI initiatives in health organizations. Several strengths and limitations should be mentioned. First, this review focused on the emergence and development stages of the innovation process, and not its implementation (despite findings of this scoping review briefly reporting on this phase). Given the disparate literature, it was important to create a strong foundation to understand the environment in which EDI can flourish. Innovation deployment is another topic that deserves its own review article. Second, due to its exploratory nature, the scoping review design may lack the rigour of systematic reviews; therefore, three mitigation measures were taken: including peer-reviewed articles, collaborating with a university librarian following Briggs’ steps, and using the PRISMA-ScR framework. This ensured that the identification and selection of studies was transparent and replicable. The authors are also a multi-disciplinary research team, with three experts in innovation management, public health and quality improvement in international health systems, as encouraged by Anderson et al^41^ who recognize that scoping reviews tend to cross multiple fields. Third, this scoping review offers a timely and distinctive contribution by addressing the state of knowledge and practices on EDI in the context of health organizations in diverse countries. By going beyond the use of the key word “employee-driven” such as using “bottom-up” and “employee involvement” in the search strategy, this scoping review gathered diverse studies that would have otherwise been missed. However, despite exploring seven databases, the United States was over-represented while only three studies occurred in middle-income countries, and none in low-income countries, this despite these countries’ increasing production of health innovations.^114^ One plausible explanation for their underrepresentation in this scoping study is the limitation to French and English languages in our search approach.

## Conclusion

 This scoping review assembles a heterogenous literature to synthesize knowledge on EDI in health organizations. It provides definitional clarity to the EDI concept, maps the conditions within which EDI initiatives flourish, and helps to understand the potential value of EDI for health organizations. This review also provides the “how-to” support for carrying out EDI in health organizations.

###  Implications for Research

 Findings from this review suggest an agenda for further research. First, innovative practices are critical given the limited budgets and resources of local government and private and publicly funded health organizations.^1^ Considering the paucity of studies focusing on hybrid and bottom-up EDI studies and their effectiveness in some contexts (eg, COVID-19), further research should explore the characteristics of this innovation process.^111^ In certain resource-constrained contexts, frugal solutions are becoming more relevant to the healthcare sector^115-117^; it would be interesting to explore how bottom-up or hybrid EDI can produce such innovations. Second, this scoping review could also be extended by exploring the processes involved in achieving EDI outcomes. Once an innovation appears to work at project level, challenges remain in supporting its spread, sustainability and scale-up.^32,97^ Third, the gap in analytical studies should be addressed to create a consensus on the definition and characteristics of EDI. Doing so would create a strong foundation on which disparate studies and theoretical insights could be gathered to inform empirical studies. Such studies will also be valuable to understand how the macro-level context such as health system policies and government bodies can be supportive of EDI. Further research could also explore how EDI can contribute to the “Quintuple Aim” of health organzations, which include the notion of health equity.^118^

## Acknowledgements

 We thank Susan Usher for providing professional writing services.

## Ethical issues

 Not applicable.

## Competing interests

 The authors declare that they have no competing interests nor conflict of interests. Regarding authors’ relationships, SBMC was a postdoctoral research fellow at Health Hub: politics, organizations and law (H-POD), the research center where JLD is the co-director. SBMC and JLD are also affiliated to the Research Center of the Montreal University Hospital Center (CRCHUM). LOD was a doctorate student at the School of Public Health of the University of Montreal where JLD is Professor.

## Supplementary files


Supplementary file 1. Search Strategy.

Supplementary file 2. Overview of the Extracted Literature.

Supplementary file 3. Overview of EDI Methods.
