# Supplementary material for: Employee-Driven Innovation in Health Organizations: Insights From a Scoping Review
Source: Int J Health Policy Manag. 2023 May 15;12:6734. doi: 10.34172/ijhpm.2023.6734 (PMC10425658; doi:10.34172/ijhpm.2023.6734)
Supplement: Supplementary file 1 — Search Strategy. [file ijhpm-12-6734-s001.pdf]

Article title: Employee-Driven Innovation in Health Organizations: Insights From a Scoping Review

Journal name: International Journal of Health Policy and Management (IJHPM)

Authors' information: Stephanie B.M. Cadeddu[1,2]*, Labante Outcha Dare[3], Jean-Louis Denis[1,2,4]

[1]Health Hub: politics, organizations, and law (H-POD), University of Montreal, Montreal, QC, Canada.

[2]University of Montreal Hospital Research Centre (CRCHUM), Montreal, QC, Canada.

[3]School of Public Health, University of Montreal, Montreal, QC, Canada.

[4]Department of Health Management, Evaluation and Policy, School of Public Health, University of Montreal, Montreal, QC, Canada

(*Corresponding author: stephanie.cadeddu@gmail.com)

Supplementary file 1. Search Strategy

Given the different terms used to discuss EDI, the concept "bottom-up" was chosen in our research equations to ensure that all the literature on innovation sourced by employees was identified. Certain studies (eg, [11,16,43]) frequently emphasized a "bottom-up approach" to innovation to describe employees' participation in innovation-related activities. This aimed to identify all body of research associated with EDI but not specifically identified as such. Also, "health organization" was favored to identify all the literature regarding health institutions. As this stage of the search process assures the rigor of a scoping review, all possible keywords pertaining to these concepts were identified, including descriptors associated with each database.

Table S1: Key words extracted from three concepts

**Concepts**	**Bottom-up**	**Innovation**	**Health organization**
**Key words**	"Bottom-up"<br>"Bottom of the corporate pyramid"<br>Frontline OR front-line<br>"Employee-driven"<br>((Participat* OR collaborat* OR involvement OR co-creat* OR co-design) NEAR/3 (worker* OR employee* OR staff))	Innovations or innovation or innovator<br>Ideat*<br>Entrepreneur*<br>Intrapreneur*<br>"Idea generation"<br>"Idea development"	(Health OR "health care" OR healthcare OR care) NEAR/3 (organisations OR organisation OR organizations OR organization OR institutions OR institution OR facility OR facilities)<br>Clinic OR clinics OR clinician* OR doctor OR doctors OR physician* OR nurse OR nurses OR patients OR patient<br>(Hospital* NOT hospitality)

Table S2: Research equations used in ProQuest Central

**ProQuest Central**<br><br>**Search strategy:** concept 1 research equations AND concept 2 research equations AND concept 3 research equations<br>**Limitations**: in title, abstract and main subject; in academic publications, books, conferences (excluded thesis and dissertation)	
**Concept 1: bottom-up** (research equations)	(ab("Bottom-up" OR "Bottom of the corporate pyramid" OR (Frontline NOT (Therap* OR treat*)) OR (front-line NOT (Therap* OR treat*)) OR "employee-driven" OR ((involvement OR Participat* OR collaborat* OR co-creat* OR co-design*) NEAR/3 (Worker* OR Employee* OR Staff*))) OR ti("Bottom-up" OR "Bottom of the corporate pyramid" OR (Frontline NOT (Therap* OR treat*)) OR (front-line NOT (Therap* OR treat*)) OR "employee-driven" OR ((involvement OR Participat* OR collaborat* OR co-creat* OR co-design*) NEAR/3 (Worker* OR Employee* OR Staff))) OR su("Bottom-up" OR "Bottom of the corporate pyramid" OR (Frontline NOT (Therap* OR treat*)) OR (front-line NOT (Therap* OR treat*)) OR "employee-driven" OR ((involvement OR Participat* OR collaborat* OR co-creat* OR co-design*) NEAR/3 (Worker* OR Employee* OR Staff))))
**Descriptors**	MAINSUBJECT.EXACT("Employee empowerment") OR MAINSUBJECT.EXACT("Employee involvement")
**Concept 2: innovation** (research equations)	(ab(Innovation OR innovations OR innovator* OR Entrepreneur* OR Intrapreneur* OR ideat* OR "idea generation" OR "idea development") OR su(Innovation OR innovations OR innovator* OR Entrepreneur* OR Intrapreneur* OR ideat* OR "idea generation" OR "idea development") OR ti(Innovation OR innovations OR innovator* OR Entrepreneur* OR Intrapreneur* OR ideat* OR "idea generation" OR "idea development"))
**Descriptors**	MAINSUBJECT.EXACT("Intrapreneurs") OR MAINSUBJECT.EXACT("Innovations") or MAINSUBJECT.EXACT("Entrepreneurs")
**Concept 3: health organization** (research equations)	(ab(((Health OR "health care" OR healthcare OR care) NEAR/3 ("organi*ation*" OR institution* OR facility OR facilities)) OR Clinic OR Clinics OR clinician* OR Doctor OR Doctors OR Physician* OR Nurse OR Nurses OR Patients OR patient OR (Hospital* NOT (hospitality))) OR ti(((Health OR "health care" OR healthcare OR care) NEAR/3 ("organi*ation*" OR institution* OR facility OR facilities)) OR Clinic OR Clinics OR clinicians OR Doctor OR Doctors OR Physician* OR Nurse OR Nurses OR Patients OR patient OR (Hospital* NOT (hospitality))) OR su(((Health OR "health care" OR healthcare OR care) NEAR/3 ("organi*ation*" OR institution* OR facility OR facilities)) OR Clinic OR Clinics OR clinicians OR Doctor OR Doctors OR Physician* OR Nurse OR Nurses OR Patient OR patients OR (Hospital* NOT (hospitality))))
**Descriptors**	MAINSUBJECT.EXACT("nurses") OR MAINSUBJECT.EXACT("Patients") OR MAINSUBJECT.EXACT("physicians") OR MAINSUBJECT.EXACT("Hospitals") OR MAINSUBJECT.EXACT("Health care")
