# Supplementary material for: Employee-Driven Innovation in Health Organizations: Insights From a Scoping Review
Source: Int J Health Policy Manag. 2023 May 15;12:6734. doi: 10.34172/ijhpm.2023.6734 (PMC10425658; doi:10.34172/ijhpm.2023.6734)
Supplement: Supplementary file 2 — Overview of the Extracted Literature. [file ijhpm-12-6734-s002.pdf]

Article title: Employee-Driven Innovation in Health Organizations: Insights From a Scoping Review

Journal name: International Journal of Health Policy and Management (IJHPM)

Authors' information: Stephanie B.M. Cadeddu[1,2]*, Labante Outcha Dare[3], Jean-Louis Denis[1,2,4]

[1]Health Hub: politics, organizations, and law (H-POD), University of Montreal, Montreal, QC, Canada.

[2]University of Montreal Hospital Research Centre (CRCHUM), Montreal, QC, Canada.

[3]School of Public Health, University of Montreal, Montreal, QC, Canada.

[4]Department of Health Management, Evaluation and Policy, School of Public Health, University of Montreal, Montreal, QC, Canada

(*Corresponding author: stephanie.cadeddu@gmail.com)

Supplementary file 2. Overview of the Extracted Literature

Table S3: Analytical studies

Authors and date	Title	Category of study & Design	Country & Focused institution(s)	Objectives/Research questions	Study key focus
Afsar et al. (2017)[49]	Do nurses display innovative work behavior when their values match with hospitals' values	2b. Conceptual and empirical (quantitative: cross-sectional design)	Thailand-public sector hospitals	To "examine how the fit between an employee's and his/her organization's characteristics impacts the employee's IWB [innovative work behaviour]" (p.158)	Enablers
Blasco et al. (2019)[50]	Incentives for public goods inside organizations: Field experimental evidence	2b. Conceptual and empirical (quantitative: natural field experiment)	USA-Massachusetts General Hospital	"To examine what drives employees' participation and effort in tasks that are above and beyond their contractual duties." (p.214)	Enablers
Brooks and Scott (2006)[51]	Nursing and Healthcare Management and Policy: Exploring knowledge work and leadership in online midwifery communication	2b. Conceptual and empirical (qualitative: case-study organizational research + interviews + naturalistic observation)	UK-non-teaching, acute hospital trust	To understand based on "knowledge work theory … how midwives can collaboratively communicate as knowledge workers to enhance knowledge creation, capture and use" (p.518)	Process
Dearmon et al. (2013)[52]	Transforming Care at the Bedside (TCAB): Enhancing Direct Care and Value-added Care	2b. Conceptual and empirical (mixed method: case-control study)	USA-urban, regional Level I trauma center	To "track and describe changes that resulted from the TCAB experience within the study hospital." (p.670)	Benefits

Fitzpatrick and Riordan (2016)[53]	Performance management of Clostridium difficile infection in hospitals the carrot or stick approach?	2a. Conceptual (literature review)	Not applicable	To "review[…] the evidence base for these policy approaches with a focus on HAI [healthcare-associated infection] and specifically CDI [Clostridium difficile infection]" (p.9)	Process and benefits
Gomes et al. (2015)[54]	The mediating effect of work engagement on the relationship between self-leadership and individual innovation	2b. Conceptual and empirical (quantitative)	Portugal-Integrated health care unit	To "explore the relationship between self-leadership, work engagement and individual innovation." (p. 1550009-1)	Enablers
Grisot et al. (2014)[55]	Innovation Of, In, On Infrastructures: Articulating the Role of Architecture in Information Infrastructure Evolution	2b. Conceptual and empirical (qualitative: case study)	Norway-Hospital	To "understand which conditions enable successful innovation in a case where technology is an II [infrastructure innovation]" (p.198)	Process and enablers
Knol and Van Linge (2009)[56]	Innovative behaviour: the effect of structural and psychological empowerment on nurses	2b. Conceptual and empirical (quantitative: cross-sectional correlational design)	Netherlands-General hospitals	To "investigate the relationship between structural empowerment/psychological empowerment and innovative behaviour" (p.361)	Enablers
Luz et al. (2019)[12]	Characteristics and behaviours of formal versus informal nurse champions and their relationship to innovation success	2b. Conceptual and empirical (quantitative: cross-sectional design using questionnaires and experts' evaluation)	Israel-Medical centers	To "identify the sociodemographic attributes, project characteristics, and champion strategies that differentiate formal and informal champions and to test these champions' distinctive success" (p.n.a.)	Enablers
Luz et al. (2019)[57]	Nursing innovation: The joint effects of championship behaviors, project types, and initiation levels	2b. Conceptual and empirical (quantitative: Cross-sectional design and multisource)	Israel-Medical centers	To "develop and test an interactionist model of project innovation in nursing" (p.407)	Enablers
Mu et al. (2018)[11]	Employee involvement in ideation and healthcare service innovation quality	2b. Conceptual and empirical (quantitative : online questionnaire survey)	Netherlands-Hospitals, medical centers, and group practices	To "identify the effects of frontline employee involvement in idea generation and of top management involvement in idea application on service innovation quality in the healthcare sector" (p.3)	Enablers
Vázquez-Calatayud et al. (2017)[58]	Transforming care in nursing: a concept analysis	2a. Conceptual	Not applicable	To "analyz[e] the concept of 'Transforming care' in nursing" (p.4) and to "identify[…] its uses, antecedents, attributes, consequences and empirical referents" (p.16)	Definition, processes, enablers and benefits
Watanabe et al. (2015)[59]	A Technology-Assisted Design Methodology for Employee-Driven Innovation in Services	2b. Conceptual and empirical (qualitative: interviews, data analysis and workshop)	Japan-Wakoen care home	To "propose a technology-assisted design methodology for employees and conduct a case study to clarify its effectiveness for employee-driven innovation" (p.6)	Process

Yasir and Masid (2019)[60]	Boundary integration and innovative work behavior among nursing staff	2b. Conceptual and empirical (quantitative: cross-sectional design)	Pakistan-Public sector hospitals	To "investigate the impact of boundary integration (BI) on innovative work behavior (IWB) of nursing staff" (p.3)	Enablers
Zuber and Moody (2018)[39]	Creativity and Innovation in Health Care Tapping into Organizational Enablers Through Human-Centered Design	2b. Conceptual and empirical (qualitative: workshop employing an empathy map and semi-structured questions)	USA-Kaiser Permanente	To "identify enabling conditions that support frontline nurses in their attempts to behave as champions of innovation and change" (p.65)	Enablers

Table S4: Descriptive studies

Authors and date	Title	Category of study & Design	Country & Focused institution (s)	Objectives/Research questions	Study key focus
Allen et al. (2019)[61]	Executive-level reviews of seclusion and restraint promote interdisciplinary collaboration and innovation	1b. Descriptive	USA-New Hampshire Hospital	To “describe[…] the positive impact of developing a forum for “witnessing” instituted at the New Hampshire Hospital” (p.261)	Process
Altman and Brinker (2016)[24]	Nursing social entrepreneurship leads to positive change	1a. Descriptive	Sweden-Medical Center First Hill	To “discuss[…] how the concept of social entrepreneurship may lend support to clinical nurses leading sustainable change initiatives” (p.30)	Process
Amador et al. (2016)[62]	Evaluation of an organisational intervention to promote integrated working between health services and care homes in the delivery of end-of-life care for people with dementia — understanding the change process using a social identity approach	1.c Evaluative (Mixed method study)	England-Care homes	To “report[…] on the qualitative component of a mixed method study aimed at evaluating an organisational intervention shaped by Appreciative Inquiry ” (p.1)	Process
Bay and Carrier (2017)[63]	L’aménagement de l’espace dans les établissements de santé : une expérience d’innovation sociale hospitalière	1a. Descriptive	France-Hôpitaux universitaires de Strasbourg (HUS)	“To take stock of the methods experimented by La Fabrique de l’Hospitalité in recent years and the results obtained.” (our translation) (p.28)	Process
Bourdon (2014)	Frontline Innovations: Implementing evidence-based care for patients with obstructive sleep apnea: The Official Voice of Perioperative Nursing	1a. Descriptive	USA-Midwest Orthopedic Specialty Hospital	To “share [team members]… experience implementing evidence-based care for patients with obstructive sleep apnea (OSA) in the perioperative setting” (p.C5)	Process
Brown (2008)[65]	Design thinking	1a. Descriptive	USA-Kaiser Permanente	To present the case of “Kaiser[‘s] Garfield Innovation Center” (p.88)	Process
Casati et al. (2010)[35]	From actors to authors — a first account about the involvement of patients in the informed consent governance of a major 4talian translational research hospital	1a. Descriptive	Italy- Ed Dardanell Heart and Vascular Center	To examine a foundation’s investment in “participatory action to promote good practice of informed consent” (p.231)	Process
Dearmon et al. (2015)[66]	Bridge to shared governance: Developing leadership of frontline nurses	1c. Descriptive (qualitative study, participant observation)	USA-Hospital with level I trauma	To “describe the process used to build leadership capacity of frontline nurses … engaged in change processes to resolve interdisciplinary operational failures” (p.70)	Enablers and benefits
Donahue et al. (2008)[67]	Nurturing Innovation in the Critical Care Environment: Transforming Care at the Bedside	1a. Descriptive	USA-UPMC Shadyside	To “discuss [the] implementation of the TCAB project at UPMC Shadyside as the vehicle to nurture care delivery innovation across settings in acute and critical care” (p.465)	Enablers and benefits

Dubrovsky et al. (2016)[68]	"We should talk" — Moving knowledge into action by learning to engage patients, families, and healthcare staff to communicate for patient safety	1a. Descriptive	Canada-Montreal Children's Hospital	To "outline[…] the development and implementation of the We Should Talk Campaign launched in October 2015" (p.141)	Process
Edwards et al. (2012)[69]	Redesigning the response to patients with trauma	1b. Descriptive and evaluative	Australia-Westmead Hospital	To "launch[…] a redesign project to identify why these problems were arising and how they could be solved" (p.31)	Process
Eines et al. (2019)[70]	Discourse analysis of health providers' experiences using service design	1c. Evaluative (Discourse analysis based on participants' experience)	Norway-Nursing home	To "explore nurses and nurse assistants' experiences using service design in a nursing home" (p.85)	Process and enablers
Eines and Vatne (2018)[71]	Nurses and nurse assistants' experiences with using a design thinking approach to innovation in a nursing home	1c. Evaluation (Qualitative)	Norway-Nursing home	To "evaluate nurses' and nurse assistants' experiences of using a design thinking approach in a nursing home in Norway" (p.429)	Process and enablers
Fieldston et al. (2016)[72]	Developing the Capacity for Rapid-Cycle Improvement at a Large Freestanding Children's Hospital	1b. Descriptive	USA-Children's Hospital of Philadelphia	To "design and pilot a quality improvement model that would engage frontline staff and ultimately lead to measurable improvements in patient- and hospital-level outcomes" (p.445)	Process
Garcia (1996)[73]	Moving Change Through the System: A Model for Staff Involvement	1a. Descriptive	USA-Beth Israel Medical Center	To "describe[…] how [c]areful development of a staff committee leads to a creative approach to problems" (p.n.a.)	Process
Garci et al. (2011)[74]	Advancing Innovation in Health Care Leadership	1a. Descriptive	USA-Mercy Gilbert Medical Center	To examine "one hospital's collaborative approach to meet the increasing demands of nursing and other hospital leaders" (p.242)	Enablers
Gonzales et al. (2017)[75]	Caring Wisely: A Program to Support Frontline Clinicians and Staff in Improving Healthcare Delivery and Reducing Costs	1b. Descriptive	USA-University of California, San Francisco's Health	To "describe a program called "Caring Wisely"®" (p.662)	Process
Govindarajan and Ramamurti (2018) [76]	Transforming Health Care from the Ground Up	1a. Descriptive	USA-University of Mississippi Medical Center	To "look at two examples of bottom-up innovation involving a radical transformation of healthcare delivery" (p.6)	Enablers
Harvey and Sotardi (2018)[16]	Carlson's Law and the Power of Frontline Innovation	1a. Descriptive	USA-Massachusetts General Hospital	To "explore Carlson's Law and how it can inform our approach to innovation" (p.86)	Enablers
Hasu et al. (2014)[77]	Learning employee-driven innovating: Towards sustained practice through multi-method evaluation	1c. Evaluative	Finland-Shared service centre	"…to introduce a workshop process to enhance the learning of employee-driven innovating (LEDI) and to evaluate in multiple ways the practical effects of the LEDI process" (p.310)	Process
Kerridge (2012)[78]	Leading change: 1—identifying the issue	1a. Descriptive	UK-NHS	To "help nurses at all levels develop the knowledge and skills they will need to initiate and manage change" (p.12)	Process

Knoff (2019)[25]	A call for nurses to embrace their innovative spirit	1a. Descriptive	USA-Gillette Children's Specialty Healthcare	To "share the author's experiences throughout the process of conceptualizing and operationalizing an innovation" and "obtaining a patent and taking her idea to practice" (p.n.a.)	Process and enablers
Laing et al. (2016)[79]	A quasi-experimental evaluation of performance improvement teams in the safety-net: A labor-management partnership model for engaging frontline staff	1c. Evaluative (mixed method study)	USA-Martin Luther King, Jr. Outpatient Center	To "investigate[…] if staff … who received UBT [Unit-Based Training] training reported greater increases in adaptive reserve" (p.E2)	Process and benefits
Lavoie-Tremblay (2004)[80]	Creating a healthy workplace: A participatory organizational intervention	1c. Evaluative (qualitative)	Canada-Hospital center	To "present[…] recent information on healthcare workers who have experienced a participatory organizational intervention" (p.569)	Process and enablers
Lindeman et al. (2003)[81]	Changing practice in residential aged care using participatory methods	1b. Evaluative	Australia-Residential care	To "report[…] on an action research component of a project aimed at enhancing the social and physical health and well being of residents of aged care settings" (p.23)	Process and enablers
Marra et al. (2013)[82]	Positive Deviance: A New Tool for Infection Prevention and Patient Safety	1a. Descriptive	Brazil-Hospital Israelita Albert Einstein	To discuss "the structure and the process of PD [positive deviance], and its ability to offer a space for experience discussions, changing ideas and making plans that emerge from team participation" (p.544)	Process
Meredith et al. (2018)[83]	Long-term impact of evidence-based quality improvement for facilitating medical home implementation on primary care health professional morale	1c. Evaluative (quantative)	USA-Medical center and community-based outpatient clinics	To explore whether "transformation to the patient-centered medical home model [was] associated with improved primary care practice morale … over time" (pp.2-3)	Benefits
Needleman et al. (2016) [84]	Engaging Frontline Staff in Performance Improvement: The American Organization of Nurse Executives Implementation of Transforming Care	1c. Evaluative (Mixed methods)	USA-Hospitals	To "examine the processes implemented at the unit level to conduct the improvement work and assess the effectiveness of the TCAB program using multiple data" (p.67)	Benefits
Neuwirth et al. (2012)[85]	Innovation profile: How Kaiser Permanente Uses Video Ethnography of Patients for Quality Improvement, Such as In Shaping Better Care Transitions	1a. Descriptive	USA-Kaiser Permanente	To describe the case of "Kaiser Permanente's Care Management Institute [which] adapted video ethnography to learn from the experiences of patients, family members, and caregivers" (p.1244)	Process
Owen et al. (1988)[86]	Changing visiting policy	1a. Descriptive	USA-Medical and Respiratory intensive Care Unit	To "describe[…] how a group of nurses…changed their ICU [Intensive Care Units] visitation policy with minimal resistance or transitional trauma" (p.369)	Process
Rotenstein et al. (2019) [20]	An Academic Medical Center-Based Incubator to Promote Clinical Innovation and Financial Value	1c. Evaluative	USA-Brigham Health	To evaluate "the Brigham Care Redesign Incubator and Startup Program (BCRISP), a flexible model to test, evaluate, and scale innovative care redesign proposals" (p.1)	Process and benefits
Shiparski and Autier (2013)[87]	Mentoring frontline managers: The vital force in stimulating innovation at the point of care	1a. Descriptive	Not applicable	To "offer[…] insights and practical applications while exploring the innovation keystones" (p.28)	Enablers
Small et al. (2017)[88]	How a Startup Accelerator at Boston Children's Hospital Helps Launch Companies	1a. Descriptive	USA-Boston Children's Hospital	To discuss the launch of the Innovation & Digital Health Accelerator (IDHA) and the factors that "led to score enough successes to prove that the model can work" (p.n.a.)	Process and enablers

Snell (2015)[15]	Leading the Way: Implementing a Domestic Violence Assessment Pilot Project by Public Health Nurses	1a. Descriptive	Canada-Community health centres	To “describe the process through which a group of front-line PHNs [Public Health Nurses] lead the development and implementation of a pilot project to address domestic violence within their practice areas” (p.66)	Process and enablers
Syme and Stiles (2012)[89]	Promoting nursing research and innovation by staff nurses	1b. Descriptive	Canada-Tom Baker Cancer Centre	To “discuss[…] how a research and innovation fund supported nurses and radiation therapists in their endeavor to conduct research and develop new ideas for clinical and other practices” (p.18)	Enablers
Telljohan (2010) [90]	Employee-driven innovation in the context of Italian industrial relations: The case of a public hospital	1a. Descriptive	Italy-Guastalla Hospital	To “look at the role played by various forms of participation in [the] experiences of employee-driven innovation in Italy” (p.228)	Process and benefits
Terwiesch et al. (2013)[43]	Innovating in health delivery: The Penn medicine innovation tournament	1a. Descriptive	USA-Penn Medecine	To “describe a ‘bottom-up’ approach to improve the patient experience” (p.38)	Process
Thompson et al. (2016)[91]	Planning, designing, building, and moving a large volume maternity service to a new labor and birth unit	1b. Descriptive	USA-Mercy Hospital St. Louis	To relate “key processes and roles responsible for a successful build … of a new labor and birth unit in a large volume perinatal service” (p.322)	Process
Thompson et al. (2015)[92]	Multiple sclerosis outpatient future groups: Improving the quality of participant interaction and ideation tools within service improvement activities	1b. Descriptive and evaluative (qualitative)	UK-Royal London Hospital (RLH)	To “describe… design-led methods to engage staff and patient participants in service improvement activities” (p.10)	Process
Upenieks et al. (2008)[3]	The relationship between the volume and type of transforming care at the bedside innovations and changes in nurse vitality	1c. Evaluative (mixed methods)	USA-Hospitals	To “evaluate the number and type of innovations tested on the medical-surgical units participating in TCAB and examine the relationship between the innovations tested and change in staff vitality on the units” (p.387)	Benefits
Wiechula et al. (2009)[93]	Improving the fundamentals of care for older people in the acute hospital setting: Facilitating practice improvement using a knowledge translation toolkit	1c. Evaluative (qualitative)	Australia-Tertiary acute hospital	To “explore and describe the implementation of a pragmatic KT intervention” (p.284)	Process and enablers

Table S5: Conference papers

Authors and date	Title	Category of study & Design	Country & Focused institution (s)	Objectives	Study key focus
**Eriksen and Tollestrup (2012)**[94]	Creating an innovative attitude at work	Descriptive (qualitative)	Denmark-Aalborg Hospital	To examine "the introduction of creative methods and approaches to the staff in a major public service institution" (p.n.a.)	Process
**Fukuda et al. (2015)**[95]	Text-mining of hand-over notes for care-workers in real operation: Toward an employee-driven innovation	Descriptive (qualitative)	Japan- Wakoen care home	To "report several use-cases of text-mining of hand-over messages where the employee actually designed a new service process based on the hand-over text-mining analysis" (p.36)	Process
**Grisot et al. (2013)**[96]	Staying under the radar: Innovation strategy in information infrastructures for health	Conceptual and empirical (qualitative)	Norway-IT department of a large hospital	To "present[…]the design, development, and initial use of a platform for patient-health provider communication" (p.1)	Enablers
**Lahtinen et al. (2014)**[97]	Co-designing Employee-driven Service Development Instruments for Public Health Care	Conceptual and empirical (qualitative)	Finland-Tampere University Hospital	To "present[…] an action research on co-designing employee-driven service development instruments for public health care professionals" (p.1)	Process and enablers
