# Supplementary material for: Employee-Driven Innovation in Health Organizations: Insights From a Scoping Review
Source: Int J Health Policy Manag. 2023 May 15;12:6734. doi: 10.34172/ijhpm.2023.6734 (PMC10425658; doi:10.34172/ijhpm.2023.6734)
Supplement: Supplementary file 3 — Overview of EDI Methods. [file ijhpm-12-6734-s003.pdf]

Article title: Employee-Driven Innovation in Health Organizations: Insights From a Scoping Review

Journal name: International Journal of Health Policy and Management (IJHPM)

Authors' information: Stephanie B.M. Cadeddu[1,2]*, Labante Outcha Dare[3], Jean-Louis Denis[1,2,4]

[1]Health Hub: politics, organizations, and law (H-POD), University of Montreal, Montreal, QC, Canada.

[2]University of Montreal Hospital Research Centre (CRCHUM), Montreal, QC, Canada.

[3]School of Public Health, University of Montreal, Montreal, QC, Canada.

[4]Department of Health Management, Evaluation and Policy, School of Public Health, University of Montreal, Montreal, QC, Canada

(*Corresponding author: stephanie.cadeddu@gmail.com)

Supplementary file 3. Overview of EDI Methods

Table S6: Top-down EDI methods

Components / Methods	Team-based (n=7)	Learning-based (n=10)	Digital (n=6)	No mention of learning, team, or digital components (n=11)
**Participatory (n=9)**	1. A working group was set up and used the Search conference methodology[90]	1. Learning employee-driven innovating process[77]<br>2. Participatory deliberative approach with a logic of training[35]	1. Computer-mediated communication that created a discussion forum[51]<br>2. Digital approach: workshop on text-mining from digital hand-note[95]	1. Focus group with healthcare workers in the targeted long-term healthcare unit[80]<br>2. Participatory action research[81]<br>3. Integration of various stakeholders in decision-making[91]
	1. Teaching and mentoring a "Frontline Innovation" group[66]			
**Design tool (n=7)**	-	1. Trained to use a design process[94]<br>2. Teaching Design-Thinking[65]	1. A technology-assisted design methodology via the user-driven product/activity design (UPAD) approach[59]	1. User-centered collaborative design[63]<br>2. Design Thinking[71]<br>3. Design Thinking[70]
		1. Developing a digital tool using design thinking while learning how to use the tool for innovation[97]		
**Participatory (n=1) and design (n=1)**	1. Creating "future groups" and combining focus group and design-led techniques[92]	-	-	-
**Competition-based (n=7)**	1. Multidisciplinary teams invited to apply to The Brigham Care Redesign Incubator and Startup Program[20]	-	1. Idea contest using a crowdsourcing platform[75]	1. Innovation contest[50]<br>2. Solicitation of brief innovation proposals[83]<br>3. Grant application at the Innovation & Digital Health Accelerator[88]<br>4. Grant-based method for research and innovation project[89]<br>5. Innovation tournament[43]
**Quality improvement (n=5)**	1. Seven teams were formed to use the Knowledge Translation Toolkit[93]	1. Trained to lead local improvement portfolios while following Plan-do study-act (PDSA) cycles[72]<br>2. Innovation community meeting for learning were established along the use of PDSA cycles[3]	1. Kaiser's quality improvement program using video-ethnography[85]	-

	1. Creation of Unit-Based Teams that are trained to use improvement tools[79]			
**Others (change management, social entrepreneurship) (n=2)**	1. A working group led by clinicians was set up and the process was inspired by managerial change techniques[69]	1. To innovate following the Social Entrepreneurship programme[24]	-	-

Table S7: Hybrid EDI methods

Methods \ Components	**Team-based (n=5)**	**Learning-based (n=0)**	**Digital (n=0)**	**No mention of learning, team, or digital components**
**Participatory methods (n=2)**	-	-	-	1. Regularly scheduled meetings to discuss and explore challenges[61]<br>2. Positive Deviance technique[82]
**Quality improvement methods (n=1)**	1. Hiring of a multidisciplinary team using the PDSA model[64]	-	-	-
**Indistinguishable methods**	1. Formation of a Nursing Practice Committee[73]<br>2. Formation of a multidisciplinary committee[86]<br>3. Formation of a domestic violence working group[15]	-	-	-
	1. Building a team and using evidence-based review[68]	-		

Table S8: Bottom-up EDI methods

Methods \ Components	**Team-based (n=1)**	**Learning-based (n=0)**	**Digital (n=0)**	**No mention of learning, team, or digital components**
**Quality improvement methods (n=1)**	1. Using quality improvement tools in teams[79]	-	-	-
**Indistinguishable methods**	-	-	-	1. Experimental process[56,97]<br>2. Social innovation theory[25]
